# Barriers to men’s involvement in antenatal and postnatal care in Butula, western Kenya

**DOI:** 10.4102/phcfm.v11i1.1911

**Published:** 2019-07-15

**Authors:** Fernandos K. Ongolly, Salome A. Bukach

**Affiliations:** 1 Center for Clinical Research, Kenya Medical Research Institute, Nairobi, Kenya; 2 Institute of Anthropology, Gender and African Studies, University of Nairobi, Nairobi, Kenya

**Keywords:** antenatal care, postnatal care, maternal health, cultural barriers, economic barriers, male involvement

## Abstract

**Background:**

Men have a lot of influence on their partners’ and children’s health. However, studies have shown their involvement in antenatal care (ANC) and postnatal care (PNC) is relatively low owing to several factors.

**Aim:**

To explore the barriers to men’s involvement in ANC and PNC in Butula sub-county, western Kenya.

**Setting:**

Butula sub-county, Busia county, western Kenya.

**Methods:**

A mixed methods study design, descriptive in nature, was used to collect both quantitative and qualitative data. A total of 96 men were selected to participate in the surveys. Also, four focus group discussions and four key informant interviews were conducted.

**Results:**

We found out that some men still participate in ANC and PNC despite the barriers. The perception that maternal health is a women’s domain and existence of alternative traditional maternal services were key cultural barriers. The men’s nature of work, low income and expenses incurred at ANC/PNC clinics were significant economic barriers. The lack of services targeting men, provider attitude, non-invitation to the clinic, time spent at the clinic and lack of privacy at the clinics were key facility-based barriers.

**Conclusion:**

A myriad of cultural, economic and health-facility barriers hinder men from active involvement in ANC and PNC. Awareness creation among men on ANC and PNC services and creating a client-friendly environment at the clinics is key in enhancing their involvement. This should be a concerted effort of all stake holders in maternal health services, as male involvement is a strong influencer to their partners’ and children’s health outcomes.

## Introduction

Antenatal care (ANC) and postnatal care (PNC) have been used for a long time as a strategy for reducing maternal and infant mortality in the promotion of safe deliveries and proper maternal and child health care. Previous studies have reported that male involvement in these services has been hindered by several factors despite the fact that they are key stakeholders in maternal and child health services.^1,2,3,4^ Key factors include the perception that maternal health issues are women matters which is largely an influence of their culture. In most African cultures, pregnancy and delivery are regarded as the domain of women, and so most men are culturally excluded from accompanying their partners to the ANC and PNC clinics.^3,5,6^ In addition, previous studies have also noted that services at the ANC and PNC clinics exclude men from active participation, thereby denying them an opportunity to learn about maternal and child health.^2,5,7^

Health care providers encourage men to actively take part in ANC and PNC; this is partly because in most cultures men influence most decisions made within their families including those concerning the health of their family members. Such decisions as to when, where and how women and children access health care are often made by men. This is influenced by men’s positions as providers and decision makers in their households. This has eventual consequences on women’s health-seeking behaviour, as it promotes early interventions leading to good maternal health and the prevention of complications during pregnancy and postnatally.^8,9^ The need for involving and encouraging men to take responsibility for their sexual and reproductive behaviour was discussed in 1994 at the international conference on population in Cairo and in 1995 at the Fourth World Conference on Women in Beijing. In these conferences, it was noted that men are in a position to change attitudes and practices through their positions as heads of homes, as well as religious and community leaders, and therefore have a lot of influence on maternal health. In addition, because they are fathers as well as husbands, they should take responsibility for reproductive health issues.^10^

As much as men may be willing to actively take part in ANC and PNC, other factors such as their lack of knowledge of the complications associated with delivery and the view that pregnancy is not a disease, high fees charged at the health facilities and uncooperative health workers are known to contribute to their low involvement.^2,4,11,12^ Addressing these could greatly improve men’s involvement in ANC and PNC. This would allow men to support their wives to prepare for delivery, and seek out appropriate care where necessary.^13^ Efforts centred on sexual and reproductive health have tried to bring men on board as participants. Services such as family planning and prevention of mother-to-child transmission (PMTCT) of HIV have seen men being included in maternal health issues. If the same is replicated to ANC and PNC, it may accrue positive results. In Kenya, there is a reported low involvement of males in ANC and PNC.^14,15^ Their involvement may help reduce maternal and infant mortality as well as improve uptake of and adherence to ANC and PNC services by women. We conducted this study to establish the barriers to men’s involvement in ANC and PNC in Butula sub-county, western Kenya.

The aim of the study was to explore the barriers to men’s involvement in ANC and PNC in Butula sub-county, western Kenya. In this regard, the following three specific objectives were explored:

- To establish the cultural barriers to men’s involvement in ANC and PNC
- To determine the economic barriers to men’s involvement in ANC and PNC
- To establish the health-facility barriers to men’s involvement in ANC and PNC.

## Research methods and design

### Setting

This study was conducted in Butula sub-county, Busia county. Butula sub-county is located in the southern part of Busia county, western Kenya. It covers a total area of 247.1 square kilometres and has a population of 121 870 people, with males constituting 47% and females constituting 53% of the population (Kenya National Bureau of Statistics, 2013). The majority of the people living in Butula are Abamarachi (A sub-tribe of the Luhyia community); however, there are also other tribes within the county that are engaged in various businesses. According to the 2013–2017 Busia County Integrated Development Plan, the infant mortality rate in the entire county was 84/1000, neonatal mortality rate was 24/1000, post-neonatal mortality rate was 41/1000, child mortality rate was 65/1000, the under 5 mortality rate was 149/1000, whereas maternal mortality rate was 41/100?000 by the year 2010. In addition, by 2010, the immunisation coverage of children under 5 years was at 95.0% and ANC compliance was at 91.5%; however, it reported only 27.3% facility-based delivery and only 20.0% male involvement in ANC and PNC.^16^

### Study design

The study design was mixed methods and descriptive in nature focussing on collecting both quantitative and qualitative data.

### Study population and sampling strategy

The study population consisted of married men of the Butula sub-county who had had children in the past 1 year or earlier at the time of the inception of the study, lived with their spouses in the same household and were above 18 years of age. Ninety-six men (24 per location) were recruited from four different *locations* (small administrative units within the sub-counties) using the simple random sampling method to take part in the survey. This was done by identifying a maternal health clinic as a reference point and randomly picking households around it from which potential participants were recruited. In the event that the selected homestead did not have an eligible participant, the research team moved to the next random homestead until they found an eligible participant. Forty men who have at least had children in the past 1 year were thereafter picked and put in groups of tens to participate in focus group discussions (FGDs).

### Data collection

Data were collected both quantitatively and qualitatively. Mixed methods research gave the freedom of triangulating and validating data from different methods. Quantitative surveys were important in finding the frequency of male participation in ANC and PNC, whereas qualitative methods provided the opportunity for contextualisation of the data collected. To collect quantitative data, structured interviews were administered using pen and paper questionnaires to 96 male participants at the household level. Questions probed their level of involvement in their partners’ last pregnancy as well as post-delivery and queried on some pre-identified barriers based on existing literature. The completion rate of the questionnaires was 100%. On the other hand, to collect qualitative data, four FGDs were conducted in different locations each involving 10 male participants where the discussions generally focussed on what stops men from being actively involved in ANC and PNC. All discussions were audio-recorded and detailed notes were also taken. Lastly, four key informant interviews (KII) involving health care workers in charge of maternal health services at selected clinics were conducted. This was also recorded in a digital recorder and detailed notes were taken. The audio files were downloaded from the recorders and uploaded in a password protected folder on the study computer, whereas the notes were kept alongside the 96 completed questionnaires in secure cabinets.

### Data analysis

Quantitative data collected from the household surveys were entered in IBM-SPSS version 20.0, cleaned, analysed and presented in the form of percentages, whereas qualitative data from the FGDs and the KII were transcribed, translated where necessary, summaries were made out of each set of data, coded thematically and presented verbatim alongside themes arising from the data.

### Ethical considerations

Ethical approval was obtained from the KNH-UoN Ethics and Research Committee (P392/07/2017) and research permission was obtained from the National Commission for Science, Technology and Innovation (NACOSTI). Informed consent was sought from all participants, and only those willing to sign a consent form were recruited for the study. All study participants were assured of confidentiality of the information that they provided and were briefed on the purpose of the study at the start of all interviews. Anonymity and privacy for the respondents were maintained throughout and the respondents were assured that their names would not be divulged at any point of the study.

## Results

### Socio-demographic characteristics

The study conducted 96 interviews at the household level in four different locations that were randomly selected, namely, Marachi east (24), Marachi central (24), Marachi north (24) and Elugulu (24).

#### Age

The respondents’ age ranged from 20 to 83 years, with an average of 38 years, and 61% of them were below the age of 40 years.

#### Education

Of the 96 respondents, 58.3% had attended school up to primary school, 32 (33.3%) had secondary education, seven (7.3%) had been to university/college, whereas one (1.1%) had never attended school.

#### Occupation

Of the 96 respondents, 60.1% were farmers while 10.5% were in formal employment. The remaining 16.8% were self-employed while 12.6% were casually employed.

#### Number of children

The number of children per respondent was also investigated: 53.1% (51) had 0–3 children, 26 (27.1%) had 4–6 children, 10 (10.4%) had 7–9 children and 9 (9.4%) had more than 10 children.

### Male involvement in antenatal care and postnatal care

The level of male involvement in ANC and PNC was assessed using the variables in Table 1. Of the 96 men interviewed, 55.8% of them had accompanied their partners to the clinic for their ANC and PNC during their last pregnancies (50% and 41.5% had joined their partners in the consultation rooms for ANC and for PNC, respectively, while only a few [33.7% at ANC and 31.6% at PNC] had joined their partners in group health discussions in the clinics). From the survey, 27.1% (at ANC) and 38.5% (at PNC) of the men interviewed reported to have never accompanied their partners to the clinic despite the fact that the majority of women (63.6% at ANC and 69.8% at PNC) had reported that their partners had attended ANC and PNC for more than two visits. Other forms of male involvement in ANC and PNC that were reported in the study included helping with household chores, offering financial support to the partner, providing food for the partner and child, and checking on partners’ and children’s health.

**TABLE 1:** Male involvement in antenatal care and postnatal care.

Activity	ANC (%)		PNC (%)	
	Yes	No	Yes	No
Accompanied partner to clinic during last pregnancy	55.8	44.2	55.8	44.2
Joined partner in consultations/counselling rooms	50.0	50.0	41.5	58.5
Joined partner in group discussions	33.7	66.7	31.6	68.4
Helped with household chores	72.6	27.4	70.5	29.5
Provided financial support	68.4	31.6	68.4	31.6
Checked on partner’s and child’s health physically	23.2	76.8	27.4	72.6
Accompanied partner for child immunisation	-	-	54.4	45.6

ANC, antenatal care; PNC, postnatal care.

#### Barriers to male involvement in antenatal care and postnatal care

Cultural, economic and health system factors were the barriers to male involvement identified by the study. These findings were outstanding in the FGD, the KII and partly from the household surveys.

### Cultural barriers

#### Maternal health issues viewed as the women’s domain

Participants of the FGD reported that they were hindered from participating in ANC and PNC as, per their culture, maternal health issues were considered the women’s domain and therefore they saw no reason to meddle in women’s issues. This was as reported below:

‘According to the Luhya culture matters concerning children are left for women Therefore, that is why some of us just choose to finance their trips to the clinics instead of joining them.’ (FGD Participant 1, Elugulu, male, 33 years)

The key informants interviewed reported that maternal health clinics have in fact been branded by the community members as *kliniki ya wamama* [women’s clinic] and *kliniki ya watoto* [infants’ clinic]. According to them, this perception discourages most men from going to those clinics which subsequently affect their active participation in ANC and PNC. This was as indicated in the following quote:

‘Some of them call the ANC clinic as pregnant women’s clinic and PNC as infants’ clinic. This makes them to only come to the health-facility when they are sick, but not to accompany their expectant wives and their infants.’ (ANC nurse, Marachi East, female)

#### Existence of alternative traditional antenatal care and postnatal care services

Men also reported the existence of alternative traditional mainstream ANC and PNC services as a barrier to their involvement in their partners’ ANC and PNC. As culturally men are excluded from some maternal and child health issues, participants reported that men were not allowed to actively take part in their partners’ ANC and PNC by traditional service providers such as traditional birth attendants (TBAs) and traditional midwives. This was reported in the following quotes:

‘We are not just supposed to rely on the clinic, because if a woman is pregnant you can also use the services of the traditional birth attendant to make sure that they are taking care of what the doctor did not do, like massaging the woman. However, when these traditional birth attendants are attending to your wife, a man is not allowed close to the place where such services are being offered. They don’t like it.’ (FGD Participant 6: Elugulu, male, 41 years)

‘They like traditional birth attendants because they are less time consuming and they come from their locality therefore making services cheaper as compared to the clinic. At the same time, when the traditional birth attendants come, the men are told to leave.’ (ANC nurse, Marachi east, female)

### Economic barriers

#### Men’s nature of work

The study findings revealed that men were hindered from participating in ANC and PNC by the nature of their work. Of the 96 participants, 66.0% (at ANC) and 67.0% (at PNC) reported that there were times when their work made it impossible for them to actively participate in ANC and PNC, as most of them were farmers, self-employed and casual workers with very low incomes who found it very difficult to set aside enough time to join their partners and children at the clinic during ANC and PNC visits. In fact, when they were asked about where they were during the ANC/PNC visits of their partners to the clinics, 87.8% (at ANC) and 84.5% (at PNC) reported that they had been either at home working in the farms or at their places of employment. This is reflected in the following quotes:

‘We work in the juakali industry and we cannot leave our businesses unattended to even for a day because on that day we shall sleep hungry. So, we let them go as we are busy looking for money.’ (FGD Participant 8, Elugulu, male, 30 years)

‘Men must go out there and search for money through work, the same money is what women need while going to the clinic, in this case when we give them money, then our money represents us in the clinic. We have to look for more and there is no time to waste being idle at the clinic.’ (FGD Participant 3, Bumala ‘A’, male, 29 years)

#### Low income

The study also revealed low income to be another barrier to male involvement in ANC and PNC. Participants reported that ANC and PNC services were expensive and therefore did all they could to minimise the cost aspect. From the survey, 68.8% (at ANC) and 63.8% (at PNC) of men reported to have been unable to actively participate in their partners’ ANC and PNC owing to a lack of financial resources. It was clear that most of them opted to finance their partners’ trips to the clinics rather than accompany them and incur extra costs. This is reflected in the following quotes:

‘You can have money, but it is not enough for both of you to go to the clinic, so in this case you have just to give her the money to go to the clinic alone.’ (FGD Participant 2, Tingolo, Male, 36 years)

‘Sometimes it can be the day for my wife’s clinic and that is the time that I do not have money, in such a case, I would rather go and work to look for money than following her to the clinic. I would tell her that since the clinic is just close by, she should go as I go to look for money so that we can get something to eat at home.’ (FGD Participant 4, Bumala ‘B’, male, 25 years)

#### Costs associated with antenatal care and postnatal care

Despite the fact that maternal health services in Kenya are free in public health facilities, costs associated with seeking these services were noted by the study as a significant barrier to male involvement in ANC and PNC. In our study, 67.0% (at ANC) and 64.9% (at PNC) were discouraged from participating owing to some associated costs that they could not afford. According to them, they went to the clinics thinking that all the services were free only to be surprised by costs such as buying surgical blades, cotton wool and gloves. At the clinics, the health care providers asked them for these items citing that the government does not provide for their free maternal health care. However, in the absence of the menfolk, their partners told the health workers of their absence and still ended up getting all the services absolutely free:

‘The government says that maternal health services are free at the clinic, however, I don’t know why we are asked for some things such as gloves and cotton wools. The nurses at the clinic claim that they are not catered for in the program but when our wives go there alone and they are asked for money for such things, they always claim that we (their husbands) will bring the money therefore I have to avoid the clinic because I have a debt there and I don’t have the money.’ (FGD Participant 7, Tingolo, male, 37 years)

### Health systems barriers

#### Lack of services targeting men

Men reported the lack of services targeting them at the clinic as a major barrier to their involvement in ANC and PNC – this was 36.2% at ANC and 34.4% at PNC. They said that most of the time when they accompanied their partners to the clinics, they ended up just sitting there and waiting. The maximum they could do was to join their partners in the clinic/counselling rooms, but even there, the health care workers only actively engaged their partners, thereby making them feel left out:

‘Just to say the truth, there is nothing that men do at the clinic, some of us just go there because we want to make our wives happy and we want them to know that we love them, however to be honest there is no service at the clinic that directly targets men. Most of them, if not all, benefit only women.’ (FGD Participant 5, Tingolo, male, 27 years)

‘Sometimes when we accompany our wives to the clinic, we end up just being idle at the benches and sometimes we start hovering around the clinic. This makes us to feel like we have wasted our time. And, because we are not engaged in any services whatsoever, such things make most men to see no need of going there since they are not the ones who are going to be served.’ (FGD Participant 7, Tingolo, male, 37 years)

#### Time spent at the clinic

Of the men interviewed, 38.3% (at ANC) and 42.6% (at PNC) reported to have been discouraged from accompanying their partners to the clinics because of the time that it took for them to be seen. From the discussions, they said that services at the clinic take a lot of time which interfered with their daily activities. This was as reported below:

‘There are a lot of things that go on at the clinics that take a lot of time and that is the time that I am required to be working maybe in the farm, or in my business. Therefore time wasting could be another barrier to us.’ (FGD Participant 8, Elugulu, male, 29 years)

The KII also reported that owing to the fact that most of their clinics are understaffed, clients take long to be served and sometimes going there is a whole day’s commitment which most men may not be able to afford:

‘Being at the clinic sometimes can be very time consuming. Especially our clinics where you get there is only one nurse who is seeing both children and pregnant mothers yet there is a long queue to attend to, this means that it will take quite some time to attend to all the clients. So, men don’t like waiting in such scenarios. They choose to leave and come later when their wives have been seen.’ (ANC nurse, Marachi East, female)

#### Attitude of health care providers

Men also reported to have been treated badly by health care providers at the clinic. Some of them reported to have been asked socially uncomfortable and embarrassing questions by the providers and sometimes were not allowed to join their partners in the clinic rooms. This was as captured below:

‘At the clinic, they ask a lot of questions of which I will be embarrassed to be there while they are asking my wife, such questions like; her last menses and the last time we had sex without a condom are some that I cannot withstand as a man and therefore choose not to go there.’ (FGD Participant 9, Elugulu, male, 40 years)

‘There is a time I went to the clinic after my wife had delivered and the doctor started asking me whether I use condoms or not, I felt so embarrassed and the next time I went there I did not join my wife in the consultation room.’ (FGD participant 6, Bumala ‘A’, male, 50 years)

#### Lack of emphasis on male involvement

The findings also revealed that, considering the fact that health care workers did not emphasise the importance of male involvement in ANC and PNC and just invited them by word of mouth through their partners, most men lacked the motivation to participate. This is as reported in the following quotes:

‘There is no emphasis that we should accompany our partners to the clinic and you know I am not the one who is going to get the services, she is the one.’ (FGD Participant 10, Bumala ‘B’, male, 42 years)

‘What makes us not to accompany our wives to the clinic is that the health care workers do not insist that we should go with them. Therefore we see no big reason to bother ourselves to the clinic.’ (FGD Participant 4, Bumala ‘B’, male, 25 years)

#### Lack of privacy and space for men at the clinic

Another issue that came out as a barrier to male involvement in ANC and PNC was the lack of privacy and space for men at the clinics. Participants reported being met with embarrassing moments at the clinics. Men reported seeing sessions that they considered private in clinic rooms. Owing to this embarrassment, they tend not to go back to the clinics in order to avoid it from happening again:

‘There is a time I also went to take my child for immunisation, I found another woman had delivered on the floor and defecated all over, I was so ashamed to be there at that particular moment. So such kinds of things have to be looked at.’ (FGD Participant 8, Tingolo, male, 43 years)

In addition, men considered the ANC and PNC clinic as spaces for women hence they were not comfortable being there. This is as reported in the following quote:

‘Sometimes you just going to the clinic make you feel like you are in the wrong place; you will find a lot of women there. It is like wearing a cloth that does not fit you. Why should you go there just to sit yet there is nothing for you?’ (FGD Participant 9, Tingolo, male, 48 years)

## Discussion

Our study sought to determine the cultural, economic and health-facility-based barriers to male involvement in ANC and PNC. Cultural barriers are very evident in our findings where men reported being discouraged from attending ANC and PNC owing to the fact that these activities are culturally considered as women’s domains. Previous studies on male involvement in maternal health services in Kenya, South Africa, Gambia and Tanzania also report similar findings.^6,8,9,17^ Considering that the community perceives these clinics as spaces for women and children, a lot of men are discouraged from going there. In addition, it was noted that owing to the existence of other traditional services such as those offered by TBAs where men are limited as far as their participation is concerned, some men were left out from active involvement in their partners’ seeking antenatal and postnatal services. These findings are not unique to our study but were also reported by other studies conducted in Athi River (Kenya) and Tanzania where TBAs were reported to tell the men to leave for a certain period when they were attending to their partners during pregnancy and after delivery.^14,18^ Despite these forms of exclusion from active participation, we noted that most men liked services offered by traditional service providers as they were less time-consuming and more cost-effective than formal health care services.^9,19^

Several economic factors came up as major barriers to male involvement in ANC and PNC. The most significant of these was the nature of the work of the menfolk, which most of them reported as an impediment to their active participation in ANC and PNC. Earlier studies have also reported similar findings.^6,9,14^ We noted that the nature of the work of the menfolk, which stipulated that they would get an income only if they reported for work and which also denied them the luxury of such benefits as leave days and day-offs, significantly limited their involvement in ANC and PNC.^20^ In most African cultures, men are the primary breadwinners in their families and would therefore choose to spend their time at work fending for their families rather than waiting for long hours at the clinics where for most of the time they are not involved. This therefore contributes to their lack of commitment at the clinics, which affects the way they participate in ANC and PNC.^14,18^ Another economic barrier that came up was the low income levels of men.^20^ In our study, we noted that men with low income levels lacked enough money to travel with their partners to antenatal and postnatal clinics and therefore opted to stay at home in order to cut down on the cost incurred. This is similar to studies in Uganda and Kathmandu which reported that men with a higher income had greater involvement in their partners’ ANC.^21,22^ From the findings of this study and those of our study, it is clear that as much as their presence at the clinics is important, men with low incomes would prefer their partners to go to the clinics alone as they themselves would proceed to work to earn an income. We also identified the costs associated with ANC and PNC services as another economic barrier to active male involvement. Apart from the cost of transport incurred when men accompanied their partners to the clinics for ANC and PNC, it was reported that whenever they went to the clinics, men were presented a lot of bills which they had not expected in the first place. This is similar to the findings of a 2010 study in Uganda on the determinants of male involvement in PMTCT, where men reported that they were discouraged from going to the clinics by the unexpected costs associated with seeking services during ANC.^21,23,24^ Therefore, to avoid meeting such costs which would compete with their financial obligations at home, men tend to avoid visiting the clinic.

Just like the cultural and economic barriers, we also noted that health-facility-based barriers play a key role in limiting men’s involvement in ANC and PNC.^10,24,25,26^ It is evident from this study and other previous studies that maternal health programmes have done little to involve men, a fact that has really hindered men’s active involvement in ANC and PNC.^17,20,27^ Previous studies have reported that men do not participate actively in maternal health services because they felt that most services excluded them.^12,27^ Another significant barrier that we noted in our study was their harsh treatment by health care workers. When men are met at the clinics by hostile staff, they tend to avoid going back there again, thereby affecting their involvement in maternal health services.^2,10,21,23^ Non-invitation at the clinic by health care providers was another reason that was reported by men about why they did not actively get involved in ANC and PNC. We noted that the health providers’ invitation to the clinics plays a big role in motivating men to participate in maternal health services. Previous studies in Zambia and other places have also reported active involvement among men who were officially invited to accompany their partners to the clinics. This is an indication of the key role that health care providers play in influencing men’s active involvement in maternal health.^4,17,27,28,30^ Men in our study also reported the lack of appropriate infrastructure as one of the barriers to their involvement in ANC and PNC. Most clinics are deprived of a proper infrastructure. Client privacy is compromised, and this discourages some men from going to those clinics. Studies in Uganda, Ghana, Zambia, South Africa and in the Pacific region have also identified the lack of privacy as a significant barrier to active male involvement in maternal health services.^2,10,15,29,30^

## Recommendations

In order to promote male involvement in ANC and PNC, and to address the barriers to their participation, the following strategies were recommended:

Primary health care workers should take up the active role of educating men on the importance of their involvement in ANC and PNC. They should also be sensitised on the need of accommodating men in antenatal and postnatal services and being non-judgemental while serving them, as men are key shareholders in women’s and children’s health. In addition, apart from primary health care workers, other stakeholders in the maternal health sector should also create awareness among men on those services in which they could actively get involved whenever they accompany their partners to the antenatal and postnatal clinics. On the other hand, the ministry of health should invest in better infrastructure that guarantees all clients their privacy at the clinic during service delivery and as they wait for their services. Lastly, the government should come up with policies that are all-inclusive and involve men as key stakeholders in maternal health.

## Conclusion

As much as men may be willing to take part in ANC and PNC, lack of services targeting them, cultural factors and their modes of subsistence were found to have a negative influence on their involvement and participation in maternal health services. Despite the fact that they may not be the direct recipients of these services, their understanding is crucial in determining women’s and children’s access to basic health services. This is of great importance as, in most families, they are key decision makers and would have a great influence on where, how and when their partners and children would seek health services.
